# PTEN/PI3K/Akt pathway alters sensitivity of T-cell acute lymphoblastic leukemia to l-asparaginase

**DOI:** 10.1038/s41598-022-08049-8

**Published:** 2022-03-08

**Authors:** Katerina Hlozkova, Ivana Hermanova, Lucie Safrhansova, Natividad Alquezar-Artieda, Daniela Kuzilkova, Adela Vavrova, Kristyna Sperkova, Marketa Zaliova, Jan Stary, Jan Trka, Julia Starkova

**Affiliations:** 1 CLIP (Childhood Leukaemia Investigation Prague), Prague, Czech Republic; 2 Department of Pediatric Hematology and Oncology, Second Faculty of Medicine, Charles University, Prague, Czech Republic; 3 University Hospital Motol, Prague, Czech Republic

**Keywords:** Cancer, Oncology

## Abstract

Childhood T-cell acute lymphoblastic leukemia (T-ALL) still remains a therapeutic challenge due to relapses which are resistant to further treatment. l-asparaginase (ASNase) is a key therapy component in pediatric T-ALL and lower sensitivity of leukemia cells to this drug negatively influences overall treatment efficacy and outcome. PTEN protein deletion and/or activation of the PI3K/Akt signaling pathway leading to altered cell growth and metabolism are emerging as a common feature in T-ALL. We herein investigated the relationship amongst PTEN deletion, ASNase sensitivity and glucose metabolism in T-ALL cells. First, we found significant differences in the sensitivity to ASNase amongst T-ALL cell lines. While cell lines more sensitive to ASNase were PTEN wild type (WT) and had no detectable level of phosphorylated Akt (P-Akt), cell lines less sensitive to ASNase were PTEN-*null* with high P-Akt levels. Pharmacological inhibition of Akt in the PTEN-*null* cells rendered them more sensitive to ASNase and lowered their glycolytic function which then resembled PTEN WT cells. In primary T-ALL cells, although P-Akt level was not dependent exclusively on PTEN expression, their sensitivity to ASNase could also be increased by pharmacological inhibition of Akt. In summary, we highlight a promising therapeutic option for T-ALL patients with aberrant PTEN/PI3K/Akt signaling.

## Introduction

Acute lymphoblastic leukemia (ALL) is the most prevalent pediatric malignancy, of which T-cell ALL (T-ALL) comprises about 15% of cases^1^. Despite the fact that pediatric T-ALL can be cured in approximately 80% of patients^2,3^, relapse still occurs in 15–20% of cases^4,5^ which then achieve an event-free and overall survival in less than 25%^6^. For standardized treatment, there has been a stable repertoire of cytostatic drugs used for the past several decades. l-asparaginase (ASNase) has been part of a first-line therapy since 1967^7^ and still remains crucial. Extensive clinical data in pediatric ALL support the benefit of intensive ASNase treatment compared with less intensive regimens^8–10^. Unfortunately, there are inter-individual differences in ASNase sensitivity and some patients also develop hypersensitivity, which could influence treatment efficacy since the resistance to ASNase is considered an unfavorable prognostic factor^7,11–13^.

Major advances in the understanding of genes, signaling networks and mechanisms that play crucial roles in the development and progression of T-ALL have led to the identification of key drivers of this disease. The phosphatase and tensin homolog (PTEN) is one of the most frequently-mutated/functionally-inactivated oncosuppressors in cancer^14^. PTEN is critical in preventing the malignant transformation of T-cells and its expression and functions are altered in human T-ALL^15^. PTEN is an inositol lipid phosphatase that acts as a major negative regulator of the phosphatidylinositol-3 kinase (PI3K)/Akt/mechanistic target of rapamycin (mTOR) signaling pathway^16^. This cascade plays a key role in controlling a wide range of essential cellular processes including cell proliferation, growth, survival and metabolism^16–18^.

We have previously reported that leukemia cells treated with ASNase reprogrammed their metabolism^19^. Moreover, we observed a correlation of specific metabolic parameters with the sensitivity to ASNase^20^. Here, we focused on the PTEN deletion in T-ALL and its effect on cell metabolism and sensitivity to ASNase. *PTEN* loss-of-function mutations/gene deletions are detected in approximately 20% of pediatric T-ALL cases^21,22^ and PTEN abnormalities are associated with an unfavorable long‐term outcome in some pediatric and adult T‐ALL patient cohorts^23–25^. Moreover, the PI3K/Akt pathway in T-ALL could also be affected by many other mechanisms, e.g. by PI3K and Akt mutations or by the decreased activity of PP2A^26^. Since PTEN functions as a negative regulator of Akt, whose activity promotes glucose metabolism^27^ and renders cancer cells dependent on aerobic glycolysis^28^, we pursued the connection between PTEN deletion, ASNase sensitivity (i.e. asparagine and glutamine depletion) and glucose metabolism. In the current study, we have observed that T-ALL cells with PTEN wild type (WT) are more sensitive to ASNase (compared to the PTEN-*null* cells) and that resistance to ASNase in the PTEN-*null* cells could be reversed by inhibiting Akt signaling.

## Results

### T-ALL cells sensitive to ASNase have a lower glycolytic profile compared to the resistant ones

**Figure 1:**
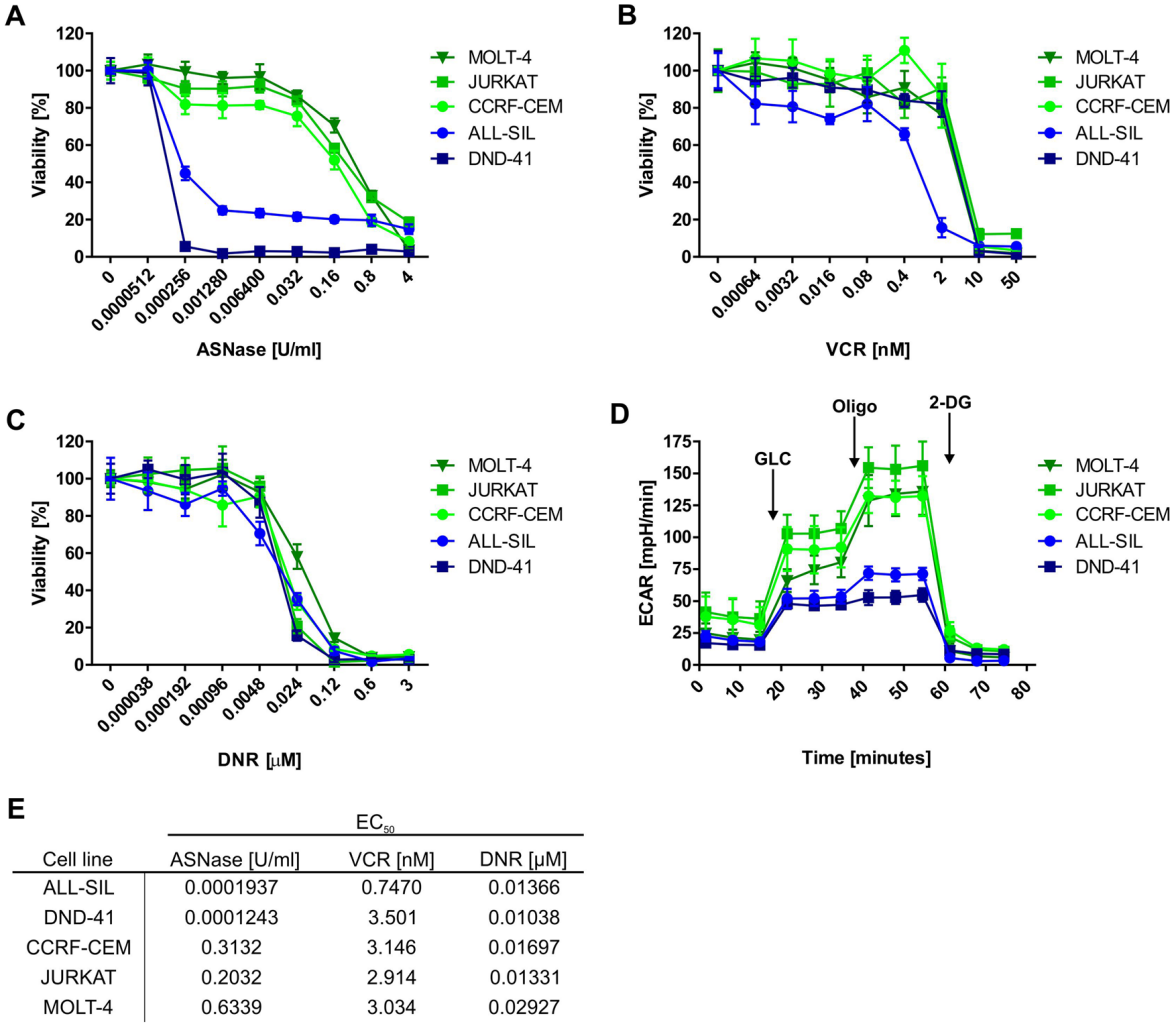
T-ALL cell lines: Sensitivity to cytostatic drugs and glycolytic profile. Sensitivity of T-ALL cell lines to (**A**) ASNase, (**B**) VCR and (**C**) DNR based on MTS assays. (**D**) Glycolytic profile of T-ALL cell lines was determined using Seahorse XFp analyzer and Glycolysis Stress Test. (**E**) EC_50_ values of ASNase, VCR and DNR in T-ALL cell lines. MTS assays were done at least in biological triplicates and six technical replicates. The Seahorse measurements were done at least in biological triplicates and six technical replicates. All the results are presented as a mean ± SD.

Current efforts in T-ALL therapy focus on relapse prevention by therapy augmentations in high-risk patients^6^. Furthermore, the search for new approaches for the treatment of recurrent disease is highly warranted. In order to find new possible therapeutic windows, we measured the sensitivity to ASNase, VCR and DNR, drugs used in T-ALL therapy, in five human T-ALL cell lines. We discovered that although their sensitivities to VCR and DNR are quite similar, the cell lines differ in their sensitivity to ASNase (Fig. 1A–C, E). ALL-SIL and DND-41 cell lines were more sensitive to ASNase than CCRF-CEM, JURKAT and MOLT-4. Since our group previously showed that the effect of ASNase is tightly connected with the glycolytic profile of leukemia cells^19,20^, we measured the glycolytic function of T-ALL cell lines on a extracellular flux analyzer. The results revealed that the two T-ALL cell lines more sensitive to ASNase have a lower glycolytic function compared to the cell lines less sensitive to ASNase (Fig. 1D). Taken together, these results indicate that high glycolytic activity might be targeted in order to increase sensitivity to ASNase in T-ALL cells.

### Identification of activated signaling pathways in T-ALL cells

**Figure 2:**
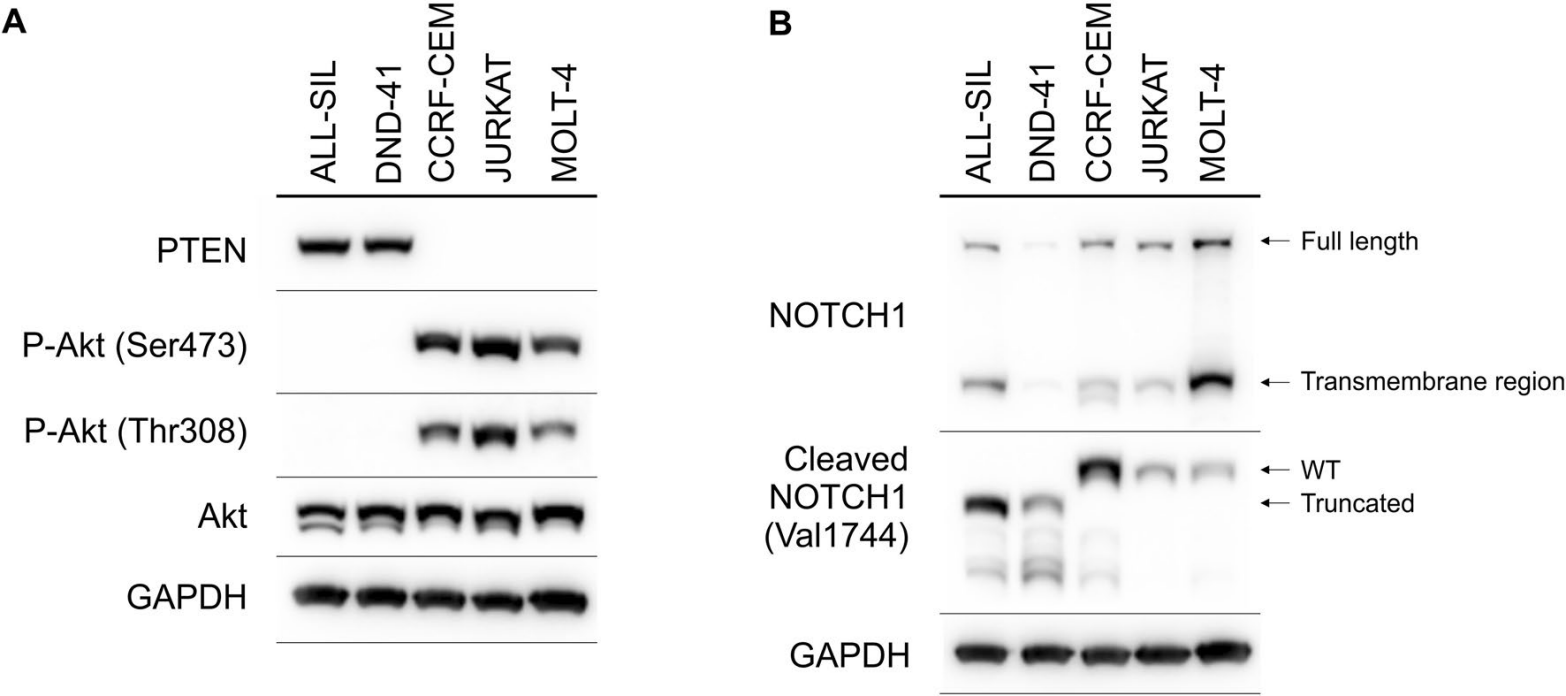
Signaling pathways in T-ALL cell lines. The immunoblot analysis of (**A**) PTEN, P-Akt (Ser473) and P-Akt (Thr308) and (**B**) NOTCH1 and cleaved NOTCH1 in T-ALL cell lines. GAPDH is shown as a loading control. Each immunoblot is a representative result of three independent experiments. Different gels are separated by lines.

Next, we tested which cellular processes could influence glycolysis and sensitivity to ASNase in T-ALL cell lines. Akt, among other pathways, regulates glycolysis^27^. Moreover, it was shown that PI3K/Akt aberrations are present in 18% of pediatric T-ALL patients^29^. Therefore, the relative activity of signaling pathways connected to the PI3K/Akt pathway were determined using western blot in five T-ALL cell lines. First of all, PTEN was detectable only in two T-ALL cell lines (Fig. 2A). In concordance with the function of PTEN, Akt was constitutively activated (P-Akt Ser473 and P-Akt Thr308) only in T-ALL cell lines without detectable PTEN (Fig. 2A). Importantly, T-ALL cell lines less sensitive to ASNase were PTEN-*null*-Akt-activated. Interestingly, we did not observe any differences in phosphorylation of some downstream targets of Akt (P-S6 and P-GSK-3β levels) between the PTEN-*positive* and the PTEN-*null* T-ALL cell lines (Fig. S1). Since NOTCH1 signaling is tightly connected with the PTEN/Akt pathway^30^, we also checked NOTCH1 and cleaved NOTCH1 (activated form of NOTCH1, NICD, NOTCH1 intracellular domain) levels in T-ALL cell lines (Fig. 2B). We detected cleaved NOTCH1 in every cell line tested. Surprisingly, ALL-SIL and DND-41, cell lines more sensitive to ASNase (and PTEN-*positive*), predominantly produced truncated NICD compared to cell lines less sensitive to ASNase (PTEN-*null*) which produced WT NICD. We did not see any difference in NOTCH1 levels between the PTEN-*positive* and the PTEN-*null* cells. Regarding c-Myc, a glycolysis activator, we did not see any significant differences between cell lines more sensitive and resistant to ASNase (Fig. S1).

### Akt inhibition enhances sensitivity to ASNase in PTEN-null T-ALL cells

**Figure 3:**
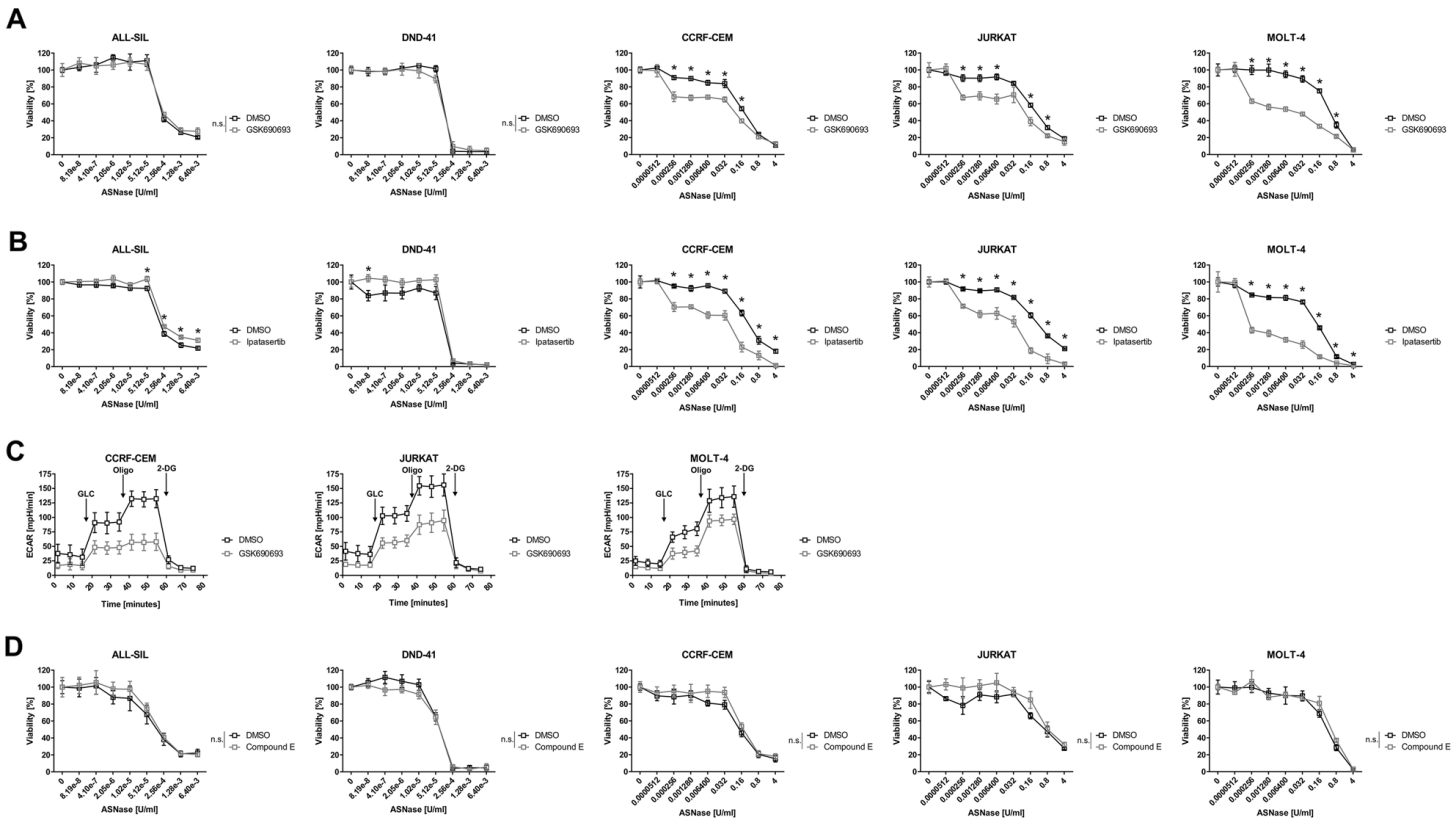
Functional study on the Akt and NOTCH1 inhibition in T-ALL cell lines. The effect of the Akt inhibitor (**A**) 1 µM GSK690693 and (**B**) 1 µM Ipatasertib on the sensitivity of ALL-SIL, DND-41, CCRF-CEM, JURKAT and MOLT-4 cell lines to ASNase. (**C**) Effect of the Akt inhibitor (1 µM GSK690693) on the glycolytic profile in CCRF-CEM, JURKAT and MOLT-4 cell lines was determined using Seahorse XFp analyzer and Glycolysis Stress Test. (**D**) The effect of the NOTCH1 inhibitor (100 nM Compound E) on the sensitivity of ALL-SIL, DND-41, CCRF-CEM, JURKAT and MOLT-4 cell lines to ASNase. Cells were co-treated with ASNase and GSK690693, Ipatasertib or Compound E for 72 h and the sensitivity to ASNase was determined based on MTS assays. All MTS assays were done at least in biological triplicates and six technical replicates. The Seahorse measurements were done at least in biological triplicates and six technical replicates. All the results are presented as a mean ± SD. * = FDR < 0.1%, *n.s.* not significant.

Since we detected higher glycolysis and a hyperactivation of Akt in T-ALL cell lines less sensitive to ASNase (Figs. 1D, 2A), we wondered whether Akt inhibition would influence glycolysis and/or sensitivity of these cells to ASNase. We used an established Akt inhibitor GSK690693 which in the concentration of 1 µM successfully inhibited the phosphorylation of Akt downstream targets S6 and GSK-3β (Fig. S2A) in both the PTEN-*null* and the PTEN-*positive* cells. Although a 72-h long cultivation with 1 µM GSK690693 influenced cell viability and proliferation (Fig. S3A), it affected both groups (PTEN-*null* and PTEN-*positive* cells) similarly. Therefore, the sole effect of the Akt inhibitor is not selective and we could test its impact on the sensitivity of T-ALL cells to ASNase. Akt inhibition significantly increased the sensitivity of the PTEN-*null*, but not the PTEN-*positive* T-ALL cell lines to ASNase (Fig. 3A). Moreover, Akt inhibition decreased glycolysis in the PTEN-*null* cells (Fig. 3C). Importantly, Akt inhibition did not change the sensitivity of neither T-ALL cells from both groups to VCR and DNR (Fig. S3C,D). These results indicate that the resistance of the PTEN-*null* cells to ASNase (but not to the other cytostatic drugs) is driven by a hyperactivated Akt and by higher glycolytic function. We also tested structurally different Akt inhibitor Ipatasertib which affected the sensitivity of T-ALL cells to ASNase similarly as GSK690693 (Fig. 3B).

As demonstrated on Fig. 2B, the PTEN-*positive* T-ALL cell lines predominantly produced truncated NICD. This is in concordance with known mutations in the NOTCH1 PEST domain in the ALL-SIL and DND-41 cell lines^31^ which generate premature STOP codons and result in truncated NICD with longer half-life than WT^32^. Previous studies speculate that cells with NICD with longer half-life are dependent on the persistently active NOTCH1 signaling^33^. Based on this information, we wanted to know whether NOTCH1 inhibition in the PTEN-*positive* T-ALL cell lines with truncated NICD influences sensitivity of these cells to ASNase. The γ-secretase inhibitor Compound E (100 nM), effectively inhibited NOTCH1 cleavage in both the PTEN-*positive* and the PTEN-*null* cells (Fig. S2B). Seventy-two-hour long cultivation of cells with 100 nM Compound E only slightly influenced the proliferation of both NICD WT and the truncated cells (Fig. S3B), meaning that in non-stressed conditions, the growth of the tested cell lines is not significantly dependent on NOTCH1 signaling. Then, we tested the effect of NOTCH1 inhibition on the sensitivity of the T-ALL cell lines to ASNase. NOTCH1 inhibition did not influence the sensitivity to ASNase in any of the T-ALL cell line tested (Fig. 3D). The results corroborate that even the cell lines with truncated NICD are not dependent on NOTCH1 signaling when treated with ASNase.

### Introduction of PTEN into JURKAT cells influences their sensitivity to ASNase

**Figure 4:**
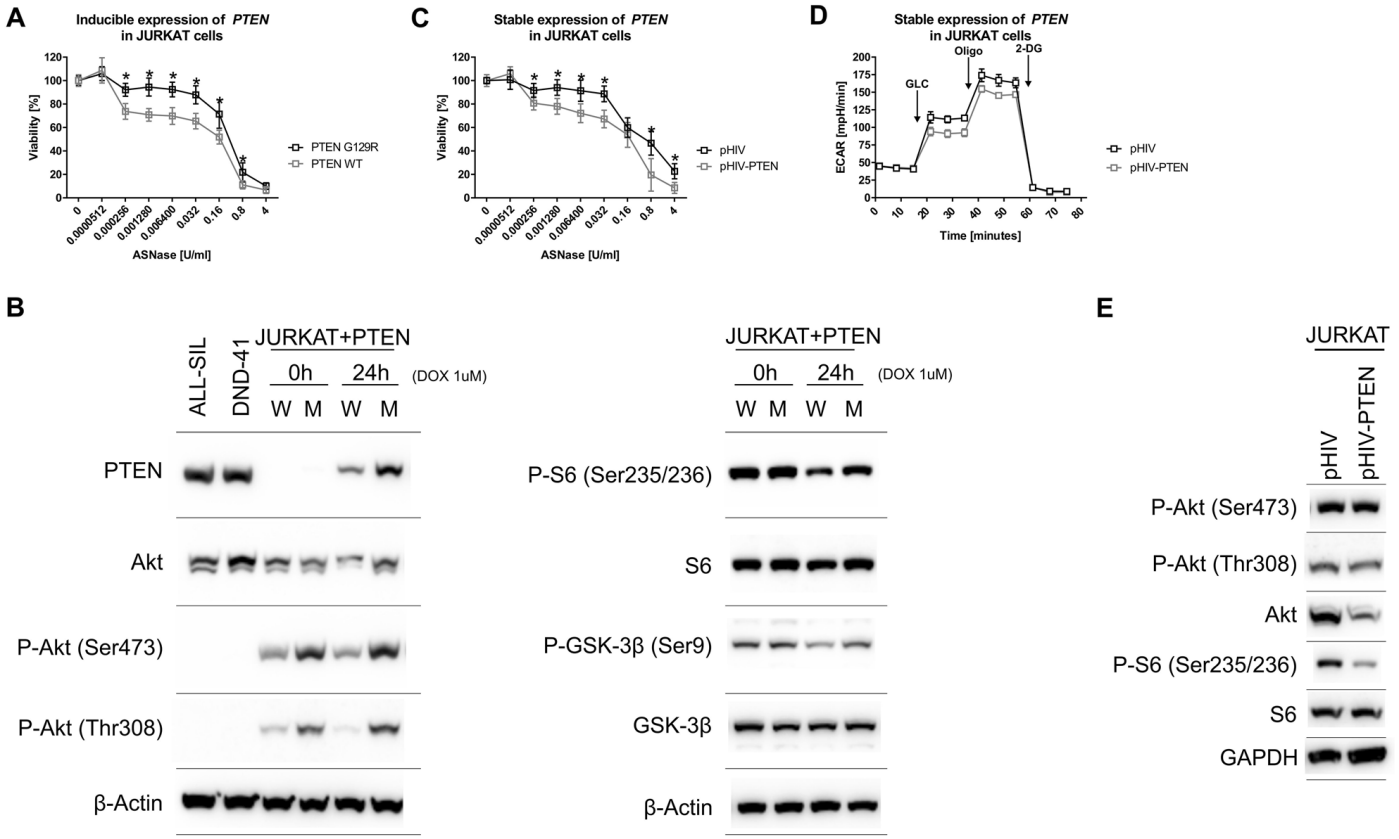
PTEN overexpression in JURKAT cells. (**A**) The effect of inducible PTEN WT and PTEN G129R expression on the sensitivity of JURKAT cells to ASNase based on MTS assays. (**B**) The immunoblot analysis of PTEN, P-Akt (Ser473), P-Akt (Thr308), P-S6, S6, P-GSK-3β and GSK-3β in JURKAT cells with inducible PTEN WT (W) and PTEN G129R (M) expression, ALL-SIL and DND-41 cell lines. β-Actin is shown as a loading control. (**C**) The sensitivity of JURKAT cells transduced with pHIV-PTEN or EV to ASNase based on MTS assays. (**D**) The glycolytic profile of JURKAT cells transduced with pHIV-PTEN or EV was determined using Seahorse XFp analyzer and Glycolysis Stress Test. (**E**) The immunoblot analysis of P-Akt (Ser473), P-Akt (Thr308), P-S6 and S6 in JURKAT cells transduced with pHIV-PTEN and EV. GAPDH is shown as a loading control. The MTS assays were done at least in biological triplicates and six technical replicates. * = FDR < 0.1%. The Seahorse measurements were done at least in biological triplicates and six technical replicates. All the results are presented as a mean ± SD. Each immunoblot is a representative result of three independent experiments. Different gels are separated by lines.

To confirm the effect of PTEN/Akt on the sensitivity of T-ALL cells to ASNase, we used an established model of inducible PTEN expression in JURKAT cells^34^. The levels of both DOX-induced PTEN, WT and G129R (PTEN without phosphatase activity), were comparable with endogenous PTEN levels in ALL-SIL and DND-41 cells (Fig. 4B). The P-Akt levels were lower in JURKAT cells expressing PTEN WT compared to PTEN G129R, which is consistent with the proper PTEN function. Noticeably, P-Akt levels are also lower in non-induced PTEN WT cells, which is probably due to the low expression of PTEN (due to a promoter leakage) which is undetectable by western blot analysis (Fig. 4B). Consistently with the function of PTEN and P-Akt, we saw diminished phosphorylation of S6 and GSK-3β proteins in JURKAT cells expressing PTEN WT compared to PTEN G129R (Fig. 4B). Next, we tested if the introduction of PTEN into JURKAT cells influences their sensitivity to ASNase. The results showed that the expression of PTEN WT, compared to PTEN G129R, sensitized JURKAT cells to ASNase (Fig. 4A). Of note, we did not see any difference in glycolysis between JURKAT cells producing PTEN WT and G129R (Fig. S4). Unexpectedly, the glycolysis in the JURKAT inducible cell model was significantly lower when compared to parental JURKAT cells. This low glycolysis is probably caused by maintaining the cells in high concentrations of G418, which has already been shown to alter mammalian cell metabolism^35^.

We also created stably transduced JURKAT cells using the pHIV-EGFP plasmid in which *PTEN* cDNA was cloned under the control of the strong EF-1α promoter (pHIV-PTEN). We saw lowered P-S6 levels in cells transduced with pHIV-PTEN compared to empty vector (EV) (Fig. 4E). This change indicates that Akt signaling is diminished. Next, we tested if the introduction of PTEN into JURKAT cells influences their sensitivity to ASNase. The results showed that introducing PTEN, compared to EV, caused JURKAT cells to be more sensitive to ASNase (Fig. 4C). Moreover, pHIV-PTEN transduction into JURKAT cells lowered their glycolysis compared to EV (Fig. 4D). Altogether, these results indicate that in T-ALL cells functional PTEN increases ASNase sensitivity through glycolysis and Akt signaling pathway modulation. Indeed, we were not able to detect PTEN in GFP-positive cells transduced with pHIV-PTEN and P-Akt levels were also unchanged compared to EV (empty vector = pHIV-EGFP, Fig. 4E). Further corroborate, the pHIV-PTEN vector expresses eGFP from the PTEN-IRES-eGFP cassette, therefore all GFP-positive cells should also express PTEN.

### Akt inhibition increases sensitivity to ASNase in some primary T-ALL cells

**Figure 5:**
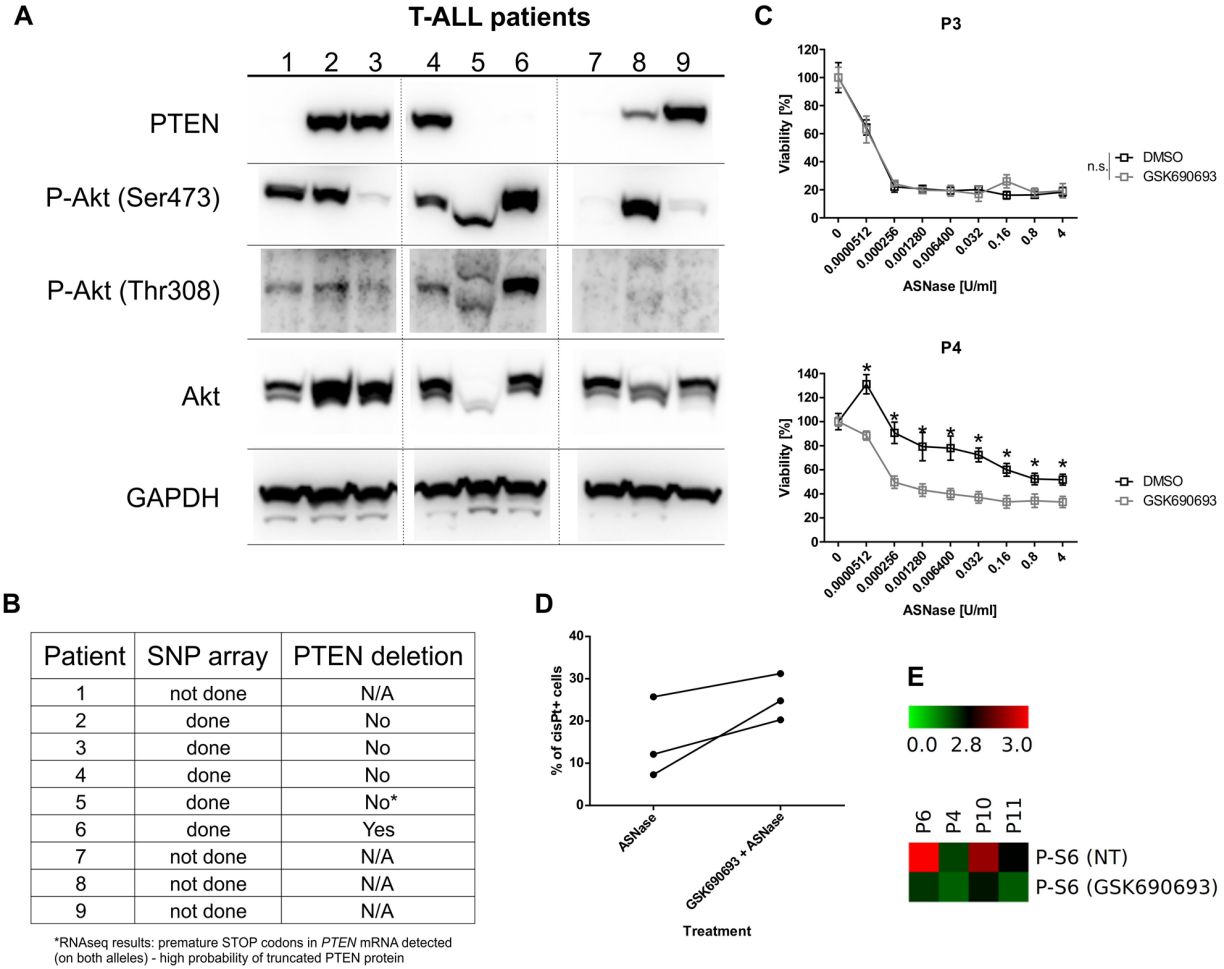
Characterization of primary leukemia cells from pediatric T-ALL patients. (**A**) The immunoblot analysis of PTEN, P-Akt and Akt in primary leukemia cells. GAPDH is shown as a loading control. Different gels are separated by lines. (**B**) SNP array analysis results from T-ALL primary cells regarding the PTEN deletion. (**C**) The effect of the Akt inhibitor (1 µM GSK690693) on the sensitivity of primary T-ALL cells to ASNase based on MTS assays. (**D**) The effect of the Akt inhibitor (1 µM GSK690693) on the sensitivity of primary T-ALL cells to ASNase based on CisPt^+^ cells detected on mass cytometry. Cells were treated for 5 h with 1 µM GSK690693 only or in combination with 0.1 U/mL of ASNase. (**E**) Phosphorylation of S6 in cells from T-ALL patients. Untreated (NT) or treated with 1 µM GSK690693 for 6 h. * = FDR < 0.1%, *n.s.* not significant.

The results from the T-ALL cell lines indicated a possible therapy target for T-ALL patients with a PTEN deletion/mutation. Therefore, we decided to examine the relative activity of the tested signaling pathways in cell lines (Fig. 2, Fig. S1) also in primary leukemia cells, which were obtained at the time of diagnosis from 9 pediatric T-ALL patients (Fig. 5A, Fig. S5A).

The patients were initially diagnosed between 1 and 20 years of age and then treated according to the INTERIM ALL 2007 (P1, 2, 6, 7, 8, 9) or the AIEOP BFM 2017 (P3, 4, 5) protocols. We did not detect PTEN using western blot in T-ALL cells from four of the patients (Fig. 5A). Moreover, in patients where single nucleotide polymorphism (SNP) array analysis could be performed, its results correlated with the PTEN protein detection (Fig. 5B). Surprisingly, P-Akt levels in patient cells negatively correlated with the presence of PTEN only in some of the patients (Fig. 5A). When we looked at the downstream Akt signaling, NOTCH1, NICD and c-Myc levels, we did not see any correlation with PTEN presence or with P-Akt levels (Fig. S5A). Next, we tested the effect of Akt inhibition on ASNase sensitivity in primary T-ALL cells (Fig. 5C, Fig. S5B). We selected three T-ALL patients with an adequate absolute number of viable cells after thawing and determined their sensitivity to ASNase. Our results showed that Akt inhibition increased the sensitivity of primary T-ALL cells to ASNase in patient P4 (higher level of P-Akt detected) but not in patient P3. Interestingly, we detected PTEN in both of the patients. These results suggest that in primary T-ALL cells, the P-Akt level is not solely regulated by PTEN. It appears that the sensitivity of primary T-ALL cells with high levels of P-Akt (regardless of PTEN presence) to ASNase could be, at least in some patients, increased by inhibiting Akt. The sensitivity to ASNase in the cells from patient P5 decreased upon Akt inhibition, which was unexpected (Fig. S5B). However, when we looked at the size of Akt and P-Akt on the western blot, it appears that Akt, P-Akt (Ser473) and P-Akt (Thr308) are mutated/truncated. Furthermore, results from RNA sequencing indicated the presence of multiple premature STOP codons in *PTEN* mRNA of patient P5. Therefore, the kinase activity of P-Akt could be disturbed.

We validated the Akt inhibition-dependent ASNase sensitivity in T-ALL primary cells from three other patients (P6, P10, P11) using Cisplatin staining on mass cytometry. Our results showed that Akt inhibition combined with ASNase treatment increased the number of Cisplatin-positive cells compared with cells treated with ASNase only (Fig. 5D). Moreover, using mass cytometry, we measured the effect of Akt inhibition of one of its downstream targets, S6, in primary cells from patients P4, P6, P10 and P11. We confirmed that GSK690693 decreased the phosphorylation of S6 in cells from all four tested patients (Fig. 5E). Importantly, our results imply the possible benefit of Akt inhibition for some T-ALL patients.

## Discussion

PTEN, whose activity antagonizes the PI3K/Akt/mTOR pathway and therefore represses tumor cell growth and survival, is one of the most frequently disrupted tumor suppressors in cancer. The significance and complexity of the PI3K signaling makes PTEN an important but challenging therapeutic target. In childhood T-ALL, PTEN deletions/mutations are quite frequent, but so far, the therapy does not reflect the possibility of the PI3K/Akt/mTOR signaling deregulation. Our results from the T-ALL cell lines gave us a fairly complex information about the part which PTEN/Akt plays in the signaling pathway. Moreover, we focused on the sensitivity of the cell lines to some of the currently used cytostatic drugs. Although the sensitivity to VCR and DNR did not vary between the five tested T-ALL cell lines, the sensitivity of these cell lines to ASNase differed dramatically. Interestingly, we could distinguish cell lines more resistant to ASNase, which were PTEN-*null* cells with constitutively activated Akt and with a higher glycolytic function, and cell lines more sensitive to ASNase, which produced PTEN, did not have phosphorylated Akt and had a lower glycolytic function. In addition, we also explored the sole influence of *PTEN* expression on ASNase sensitivity, glycolysis and the activation of Akt. Our results confirmed that introduction of PTEN into PTEN-*null* cells increased their sensitivity to ASNase and lowered their glycolytic function, both probably via the inhibition of Akt. The connection between PTEN and P-Akt levels in the T-ALL cell lines fully corresponds with the phosphatase activity of PTEN^26^. Moreover, it has been shown that Akt signaling regulates nutrient uptake and metabolism. More specifically, Akt stimulates glucose uptake^36^. Higher glycolytic function reported by us thus corresponds to higher P-Akt levels. Metabolic reprogramming in general is now considered as one of the hallmarks of cancer^37^. Therefore, targeting cancer metabolism has become an attractive therapeutic approach, one which, nevertheless requires a therapeutic window in which tumor cells have a great dependence on specific enzymes^38^. Constitutive activation of Akt in T-ALL cell lines less sensitive to ASNase inspired us to pursue Akt as a possible therapeutic target. We discovered that resistance to ASNase in T-ALL cell lines with constitutively active Akt could be decreased by the inhibition of Akt by two structurally different inhibitors. Although several Akt inhibitors are now undergoing clinical trials, only one has been evaluated in hematological malignancies to this day (GSK2110183 in multiple myeloma and chronic lymphocytic leukemia)^39^.

PTEN deletion occurs in a relevant fraction of a variety of cancers (e.g. breast or prostate), where it results in a worse prognosis and outcome^40,41^. The loss of PTEN in 20% of pediatric T-ALL cases is thus definitely worthy of attention. In this study, we discovered that in primary T-ALL the relationship between PTEN and P-Akt is not as straightforward as in cell lines. Similar ambiguous findings were reported in endometrioid cancers. In these malignancies PI3K pathway aberrations occur in more than 80% of cases and it appears that the PTEN protein loss serves as a dominant activation event of this particular pathway. However, in tumors with retained PTEN protein, PI3K pathway mutations phenocopy PTEN loss, resulting in pathway activation^42^. When we focused on Akt, we were able to increase the sensitivity to ASNase by inhibiting Akt in primary cells from T-ALL patient with high P-Akt level. Noteworthy, T-ALL cells from this patient also produced PTEN. On the other hand, Akt inhibition did not change the sensitivity to ASNase in cells with low P-Akt level. Using mass cytometry, we confirmed the increase of the sensitivity to ASNase in Akt-inhibited cells from three other T-ALL patients. Importantly, we discovered that mass cytometry presents a suitable tool for evaluating drug efficacy in frozen T-ALL samples. In summary, regardless of the presence of PTEN, the sensitivity to ASNase could be increased in some primary T-ALL cells which have constitutively active Akt. Altogether, the results of our study suggest possible improvements of T-ALL therapy for patients with higher levels of P-Akt.

## Methods

### Cell culture

Human T-cell leukemia cell lines (ALL-SIL, CCRF-CEM, DND-41, JURKAT, MOLT-4) were purchased from German Collection of Microorganisms and Cell Cultures (DSMZ, Braunschweig, Germany;). The cell lines were negative for mycoplasma contamination and were cultivated in RPMI-1640 medium with GlutaMAX™ supplemented with 10% fetal calf serum, penicillin (100 U/mL) and streptomycin (100 μg/mL) under controlled conditions (37 °C, 5% CO_2_). The cultured cells were split every two to three days and maintained in exponential growth phase. JURKAT stable clones that express the WT or mutant PTEN (G129R) under the control of the tetracycline-inducible system (Tet-on)^34^ were kindly provided by Dr. Arthur Weiss from the Howard Hughes Medical Institute (University of California, San Francisco, USA). PTEN G129R is a tumor-derived mutation that is defective in both the protein and the lipid phosphatase activity^34^. Clones were maintained in RPMI-1640 medium with GlutaMAX™ supplemented with 10% Tet system-proved fetal calf serum and 2 mg/mL G418 and 300 µg/mL hygromycin B and expression of *PTEN* was induced by doxycycline (DOX), a tetracycline analog.

### Plasmids and lentiviral infection

pHIV-EGFP lentiviral vector containing *PTEN* cDNA was used (pHIV-PTEN). *PTEN* cDNA was amplified with 5′-TATTCTAGAGTACCCGGGATGACAGCCATCATCAAAGAG-3′ and 5′-GGAGAGGGGCGGATCCCATGGTGTTTTATCCCTCTTGAT-3′ primers and cloned under the control of the EF-1α promoter into the pHIV-EGFP vector. Lentiviral particles were produced in HEK-293T cells using 30 µg of the lentiviral vector (pHIV-EGFP or pHIV-PTEN), 24 µg of the packaging vector, 2.8 µg of the envelope-plasmid and polyethylenimine. Lentiviral particles were collected from the supernatant after 48 and 72 h and concentrated using the Lenti-X Concentrator (Takara Bio, Japan). JURKAT cells were transduced using spinoculation by centrifuging the cells for 2 h at 800×*g* at RT. Forty-eight hours after transduction, GFP+ cells were sorted. Transduced JURKAT cells were maintained as GFP + cells (> 90%). pHIV-EGFP was a gift from Bryan Welm & Zena Werb (Addgene plasmid #21373; http://n2t.net/addgene:21373; RRID:Addgene_21373)^43^.

### Patient samples

Bone marrow or peripheral blood samples from untreated children initially diagnosed with T-ALL were collected from the Czech Pediatric Hematology Centers. To be able to perform the here described experiments, only patients with the blast percentage higher than 80% and with a high cellularity were included. Within 24 h after aspiration, the mononuclear cells were isolated by density gradient centrifugation using Ficoll-Paque PLUS (GE Healthcare, UK). All the samples were obtained with the informed consent of the children’s parents or guardians as well as the approval of the Ethical Committee of the University Hospital Motol, Prague, Czech Republic, study no. 201528848A. All experiments were performed in accordance with relevant guidelines and regulations. The isolated blasts were frozen in 90% fetal calf serum and 10% DMSO. After thawing, the blasts were maintained in X-VIVO™ 15 Medium with l-glutamine and gentamicin supplemented with 10% fetal calf serum, penicillin (100 U/mL), streptomycin (100 μg/mL), IL-7 (50 ng/mL) and insulin-transferrin-sodium selenite supplement (Sigma-Aldrich, St Louis, MO, USA).

### Cell survival and proliferation

To evaluate the cytotoxicity of ASNase, vincristine (VCR), and daunorubicin (DNR), MTS (dimethylthiazol carboxymethoxyphenyl sulfophenyl tetrazolium) assays were performed using a CellTiter 96 AQueous One Solution Cell Proliferation Assay (Promega Corporation, Wisconsin, USA) according to the manufacturer’s instructions. Leukemia cell lines (1.2 × 10^4^ cells) and primary cells (1–3 × 10^5^ cells) were seeded. The MTS assay was performed at least in three independent experiments.

### Seahorse extracellular flux analysis

Glycolytic functions of T-ALL cell lines were measured on the Seahorse analyzer XFp (Agilent Technologies, Inc., CA, USA) using a Glycolysis stress test. Cells were plated in XF RPMI medium pH 7.4, at a density of 100,000 cells/well in XFp tissue culture plates coated with CellTak (Corning GmbH, Wiesbade, GER) according to the Agilent Seahorse protocol for the seeding of suspension cells. Final concentrations of the injected drugs were 10 mM glucose, 2 μM Oligomycin A and 100 mM 2-deoxy glucose (2-DG). All of the Seahorse measurements were done at least in biological triplicates and three technical replicates.

### SNP array

Copy number aberrations (CNA) in the PTEN gene were screened by single-nucleotide polymorphism array (HumanOmni Express BeadChip or Infinium Global Screening Array, Illumina, USA). DNA labeling and hybridization were performed according to the manufacturer’s instructions. The GenomeStudio software v2011.1 (Illumina) was used for genotype calling and quality control. Copy number variations (CNV) were called using the CNV Partition 2.4.4 algorithm plug-in within the GenomeStudio software. The resulting data (Log R ratio corresponding to copy number and B allele frequency corresponding to SNP genotype) were visually inspected in the Illumina Chromosome Browser and the CNV calls were manually curated.

### Electrophoresis and western blotting

Protein lysates were prepared as previously described^44^. Proteins (30 μg per well) were resolved by NuPAGE Novex 4–12% Bis–Tris Gels (ThermoFisher Scientific Inc., MA, USA) and transferred onto a nitrocellulose membrane (Bio-Rad, CA, USA). The membrane was probed overnight with the primary antibodies listed in Table S1. The bound antibodies were detected with the appropriate secondary antibodies conjugated to horseradish peroxidase (Bio-Rad, CA, USA) and visualized using an enhanced chemiluminescence reagent and documented by Uvitec (Cambridge, UK).

### Mass cytometry

For evaluation of cell viability, 1 µM Cell-ID Cisplatin-198Pt (Fluidigm) was added into the cell culture. After 5 min incubation cells were washed twice in MaxPar Cell Staining Buffer (CSB, Fluidigm; 570 g, 5 min, RT). Samples were fixed with 1.6% paraformaldehyde and stored (10% glycerol in fetal bovine serum at − 20 °C). The samples were thawed, washed with MaxPar Cell Staining Buffer (CSB, 800*g*, 5 min, RT), and barcoding monoclonal antibodies (moAbs) were added (15 min, RT). The cells were washed, pooled together and washed in CSB (800*g*, 5 min, RT). To minimize technical variability between stimulated and unstimulated samples, differently treated samples from particular donors were barcoded, mixed and further processed in one tube. The samples were stained according to the MaxPar Phosphoprotein Staining with Fresh Fix (Fluidigm) protocol as recommended by the manufacturer. The instrument (Helios, Fluidigm, CyTOF 6.7.1014 software) was prepared for acquisition according to the manufacturer’s recommendation. Signal spillover between channels was corrected using the CATALYST R package as described previously^45^. Compensated fcs files were further processed using FlowJo (v10.5, FlowJo LLC). T-ALL blasts were gated as CD7^+^CD45^dim^CD3^+^CD19^-^ and homogenous population on CD4 vs CD8 scatterplot. Heatmaps were created in MeV software^46^ by displaying arcsinh transformed absolute values of median intensities.

### Statistical analysis

The statistical analysis was performed using a paired Student’s *t* test (FDR < 0.1%), EC50 was calculated using non-linear regression (variable slope method). Calculations were performed using GraphPad Prism 6 software. Group data are expressed as the mean ± standard deviation (SD).

## Supplementary Information

Supplementary Information.
