# Supplementary material for: PTEN/PI3K/Akt pathway alters sensitivity of T-cell acute lymphoblastic leukemia to l-asparaginase
Source: Sci Rep. 2022 Mar 8;12:4043. doi: 10.1038/s41598-022-08049-8 (PMC8904819; doi:10.1038/s41598-022-08049-8)
Supplement: Supplementary file 1 — Supplementary Information. [file 41598_2022_8049_MOESM1_ESM.docx]

**
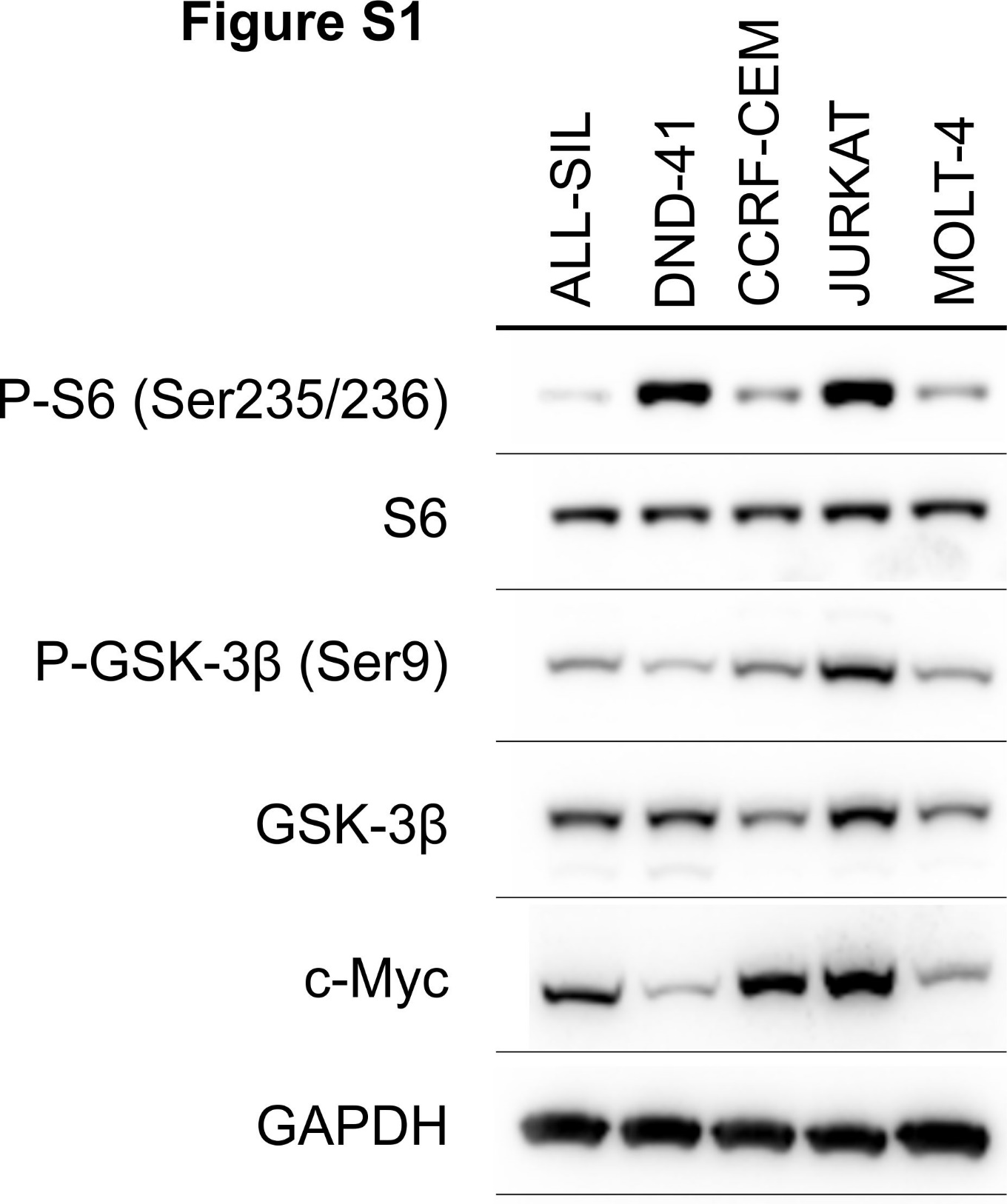
**

**Supplementary figure 1: Signaling pathways in T-ALL cell lines.**

The immunoblot analysis of P-S6, S6, P-GSK-3β, GSK-3β and c-Myc in T-ALL cell lines. GAPDH is shown as a loading control. The immunoblot is a representative result of three independent experiments.

**
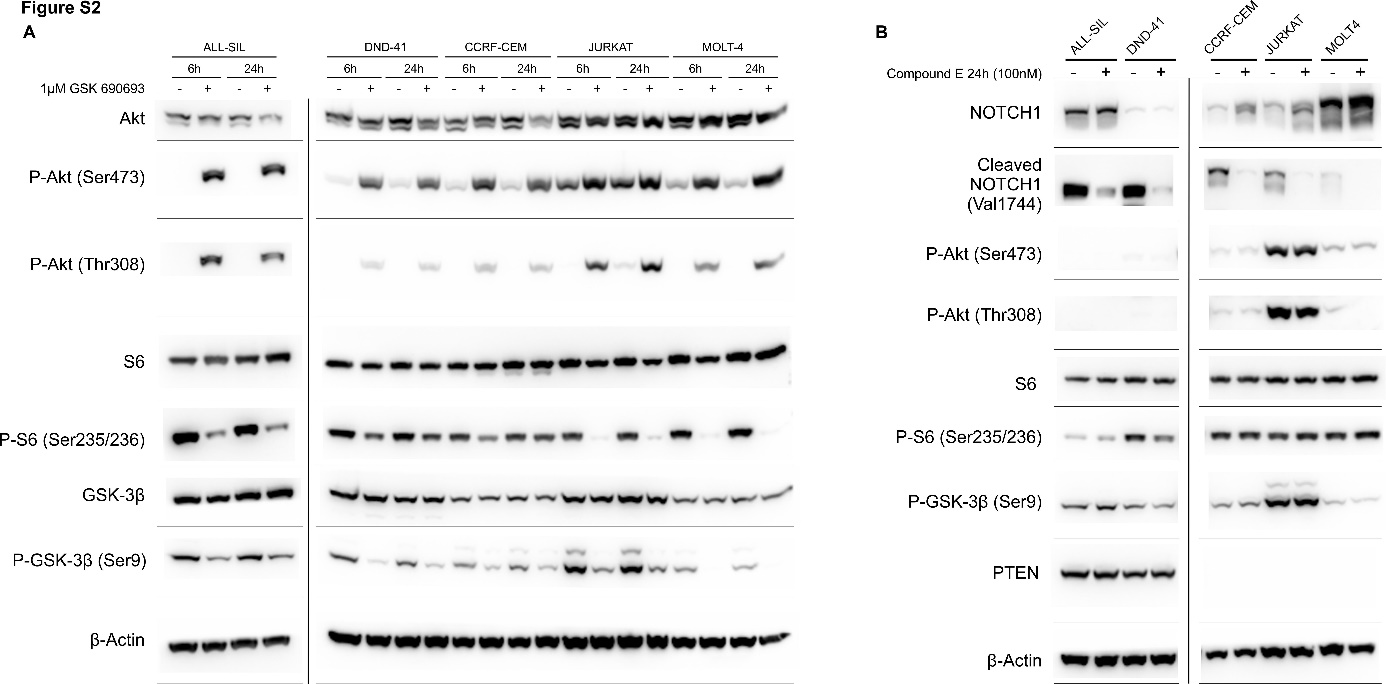
**

**Supplementary figure 2: Effect of Akt and NOTCH1 inhibition on signaling pathways in T-ALL cell lines.**

The immunoblot analysis of **(A)** 1µM GSK690693 and **(B)** 100nM Compound E effect on signaling pathways in ALL-SIL, DND-41, CCRF‑CEM, JURKAT and MOLT-4 cell lines. Β-actin is shown as a loading control. Each immunoblot is a representative result of three independent experiments.

**
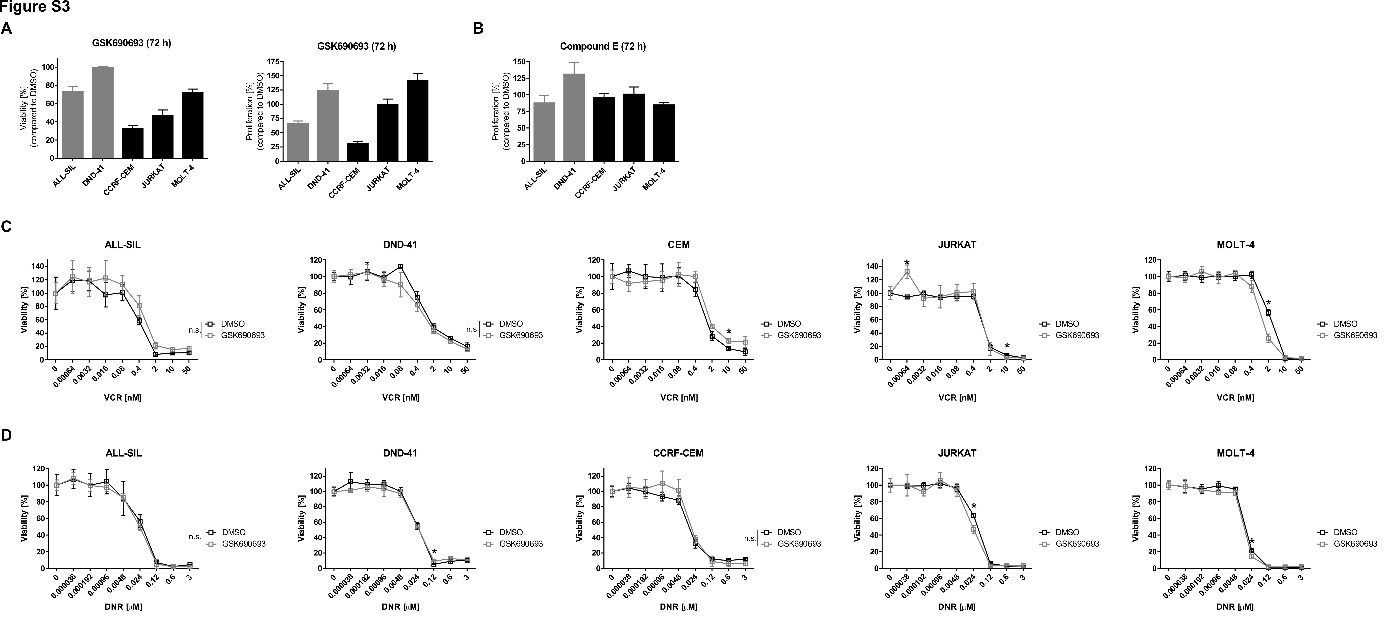
**

**Supplementary figure 3: Akt and NOTCH1 inhibition effect on the growth and sensitivity of T-ALL cell lines to VCR and DNR.**

**(A)** Effect of GSK690693 on the viability and proliferation of T-ALL cell lines. Cells were treated with 1µM GSK690693 or DMSO for 72 hours. **(B)** Effect of Compound E on the proliferation of T-ALL cell lines. Cells were treated with 100nM Compound E or DMSO for 72 hours. Viability was determined by MTS assay and proliferation by cell count. Effect of Akt inhibitor (1µM GSK690693) on the sensitivity of ALL-SIL, DND-41, CCRF‑CEM, JURKAT and MOLT-4 cell lines to **(C)** VCR and **(D)** DNR. Cells were co‑treated with VCR or DNR and GSK690693 or 72 hours and sensitivity to VCR or DNR was determined based on MTS assay. All MTS assays were done at least in biological triplicates and six technical replicates. Cell counts were performed at least in biological and technical triplicates. All the results are presented as a mean ± SD. *=FDR<0.1%, n.s.=not significant.

**
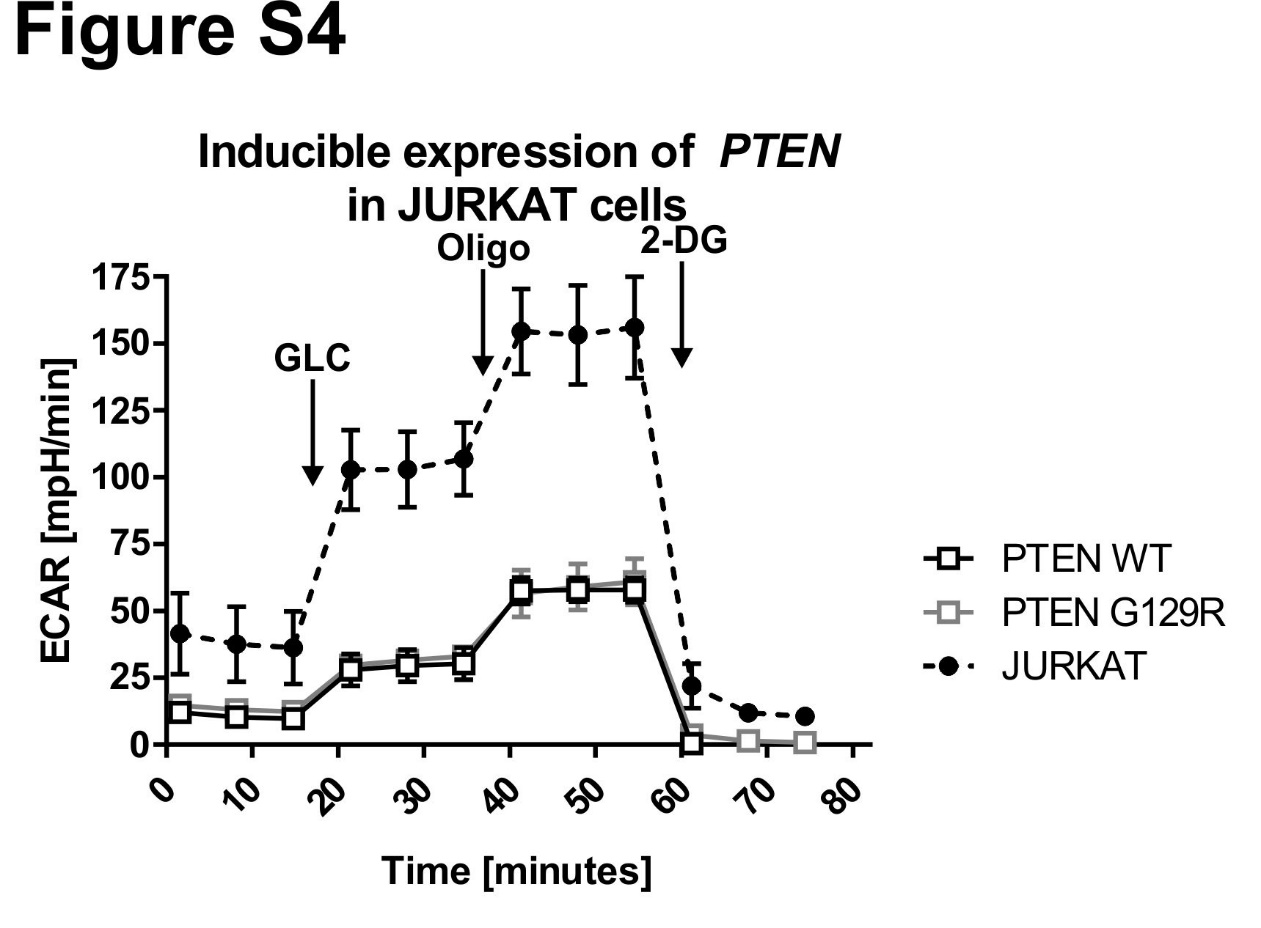
**

**Supplementary figure 4: Glycolysis in JURKAT cells with inducible PTEN expression.**

Glycolytic profile of JURKAT cells with inducible PTEN WT and PTEN G129R expression and normal JURKAT cells determined using Seahorse XFp analyzer and Glycolysis Stress Test. Measurements were done at least in biological triplicates and five technical replicates. The results are presented as a mean ± SD.

**
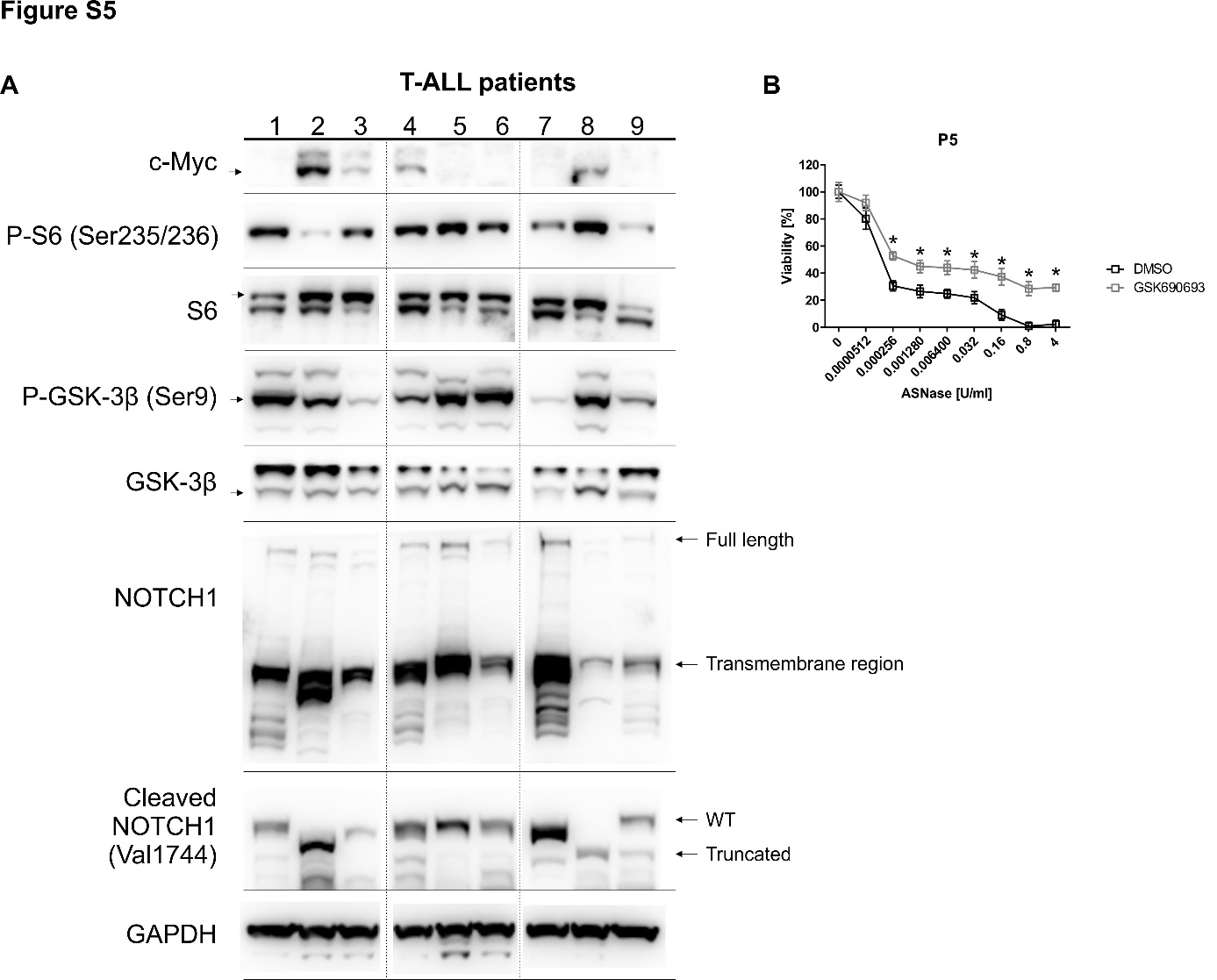
**

**Supplementary figure 5: Signaling in primary leukemia cells from pediatric T-ALL patients.**

**(A)** The immunoblot analysis of c-Myc, P-S6, S6, P-GSK-3β, GSK-3β, NOTCH1 and NICD in primary T‑ALL cells. GAPDH is shown as a loading control. **(B)** Effect of Akt inhibitor (1µM GSK690693) on the sensitivity of primary T-ALL cells to ASNase based on MTS assay. *=FDR<0.1%.

**Supplementary table 1: List of primary antibodies.**

| **Primary antibody** | **Supplier** | **Description** | **Dilution** |
| --- | --- | --- | --- |
| Akt (pan) (40D4) | Cell Signaling Technology | Mouse monoclonal Ab | 1:1000 |
| Phospho-Akt (Ser473) (D9E) XP® | Cell Signaling Technology | Rabbit monoclonal Ab | 1:1000 |
| Phospho-Akt (Thr308) (244F9) | Cell Signaling Technology | Rabbit monoclonal Ab | 1:1000 |
| S6 Ribosomal Protein (5G10) | Cell Signaling Technology | Rabbit monoclonal Ab | 1:1000 |
| Phospho-S6 Ribosomal Protein (Ser235/236) (D57.2.2E) XP® | Cell Signaling Technology | Rabbit monoclonal Ab | 1:1000 |
| c-Myc Antibody | Cell Signaling Technology | Rabbit polyclonal Ab | 1:1000 |
| GSK-3β (27C10) | Cell Signaling Technology | Rabbit monoclonal Ab | 1:1000 |
| Phospho-GSK-3β (Ser9) (5B3) | Cell Signaling Technology | Rabbit monoclonal Ab | 1:1000 |
| PTEN (138G6) | Cell Signaling Technology | Rabbit monoclonal Ab | 1:1000 |
| Notch1 (D1E11) XP® | Cell Signaling Technology | Rabbit monoclonal Ab | 1:1000 |
| Cleaved Notch1 (Val1744) (D3B8) | Cell Signaling Technology | Rabbit monoclonal Ab | 1:1000 |
| Anti-GAPDH antibody (clone GAPDH-71.1) | Sigma-Aldrich | Mouse monoclonal Ab | 1:10000 |
| β-Actin (clone AC-74, ascites fluid) | Sigma-Aldrich | Mouse monoclonal Ab | 1:4000 |


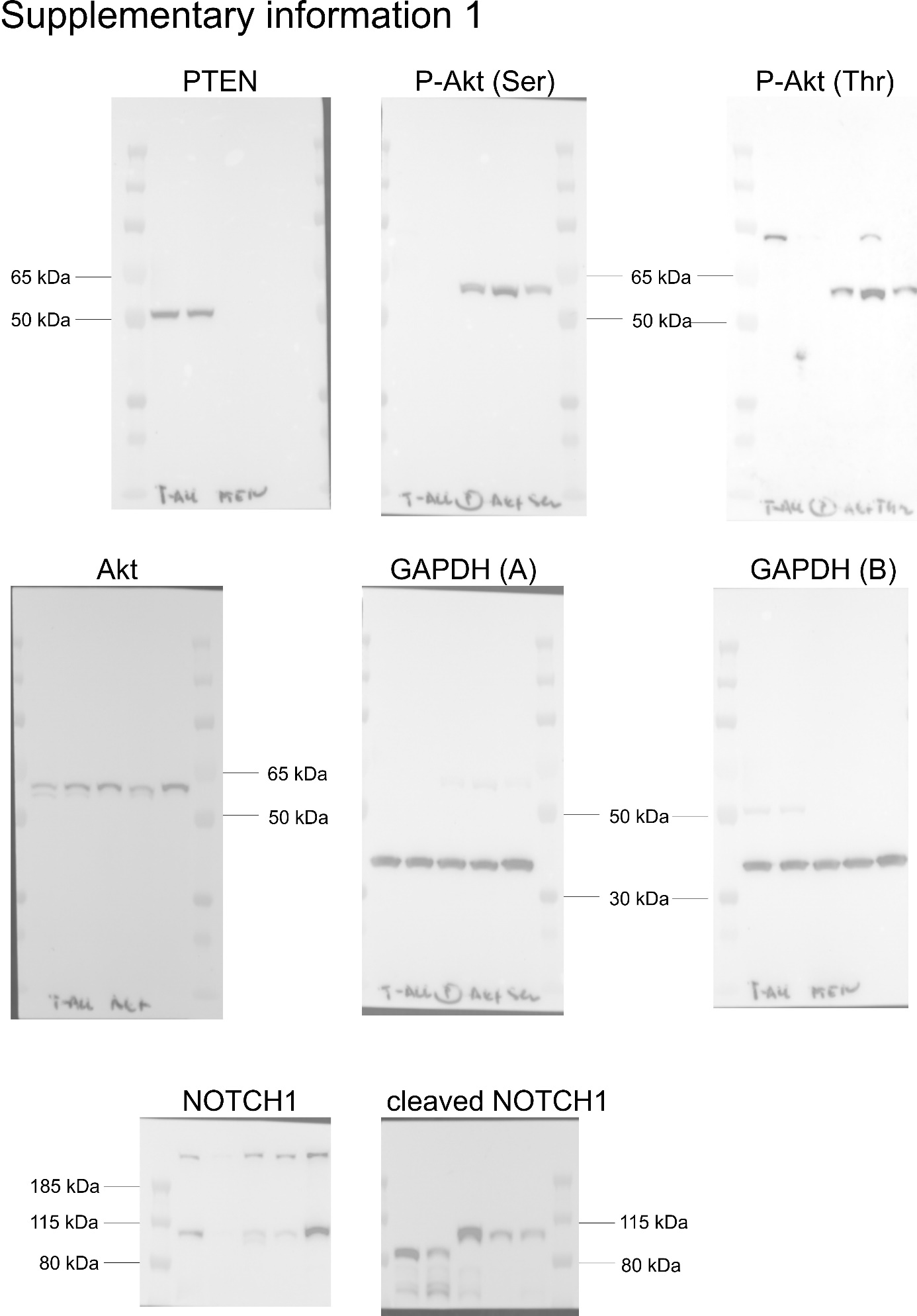


**Supplementary information 1: Full-length gels from Figure 2.**


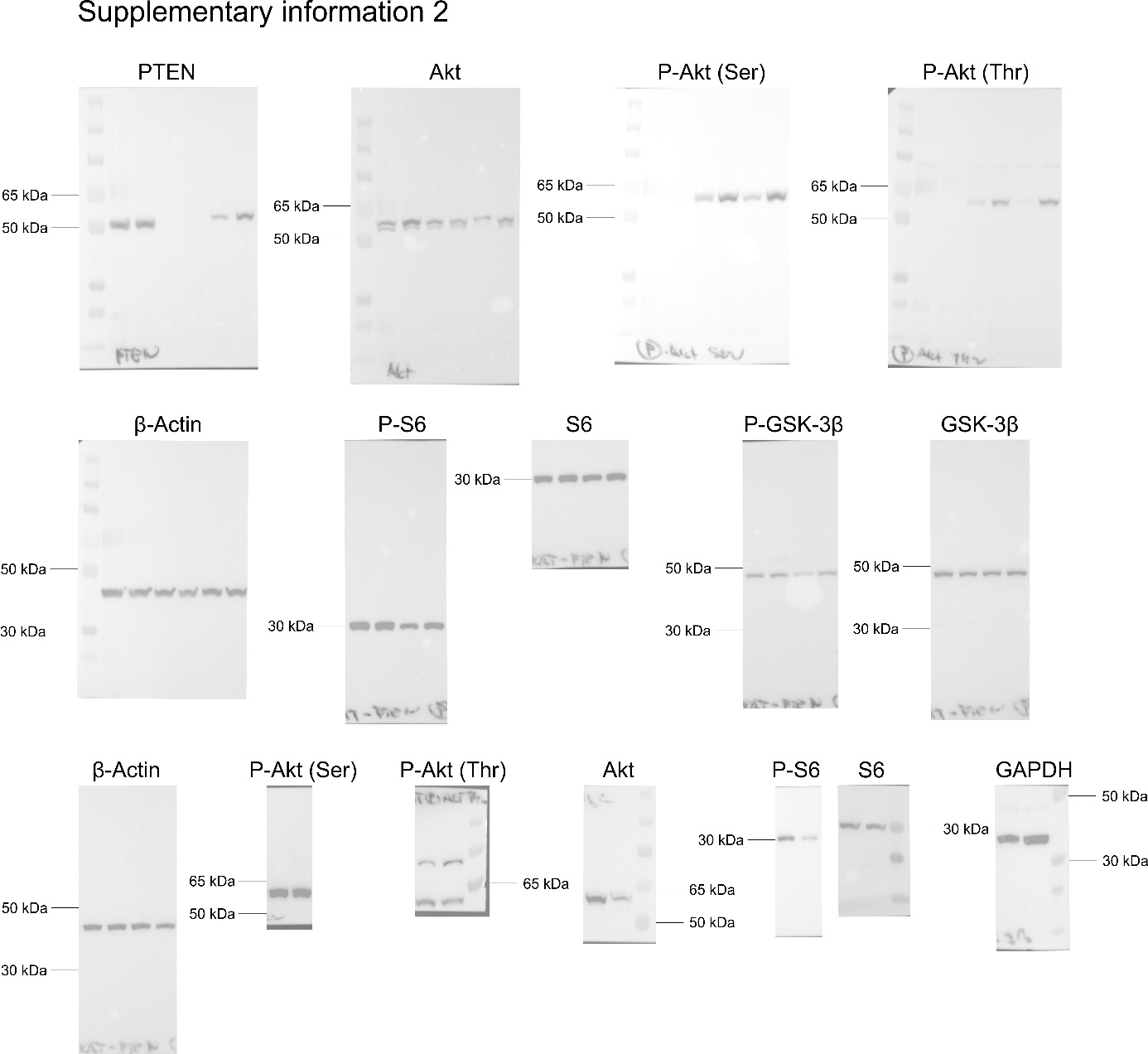


**Supplementary information 2: Full-length gels from Figure 4.**


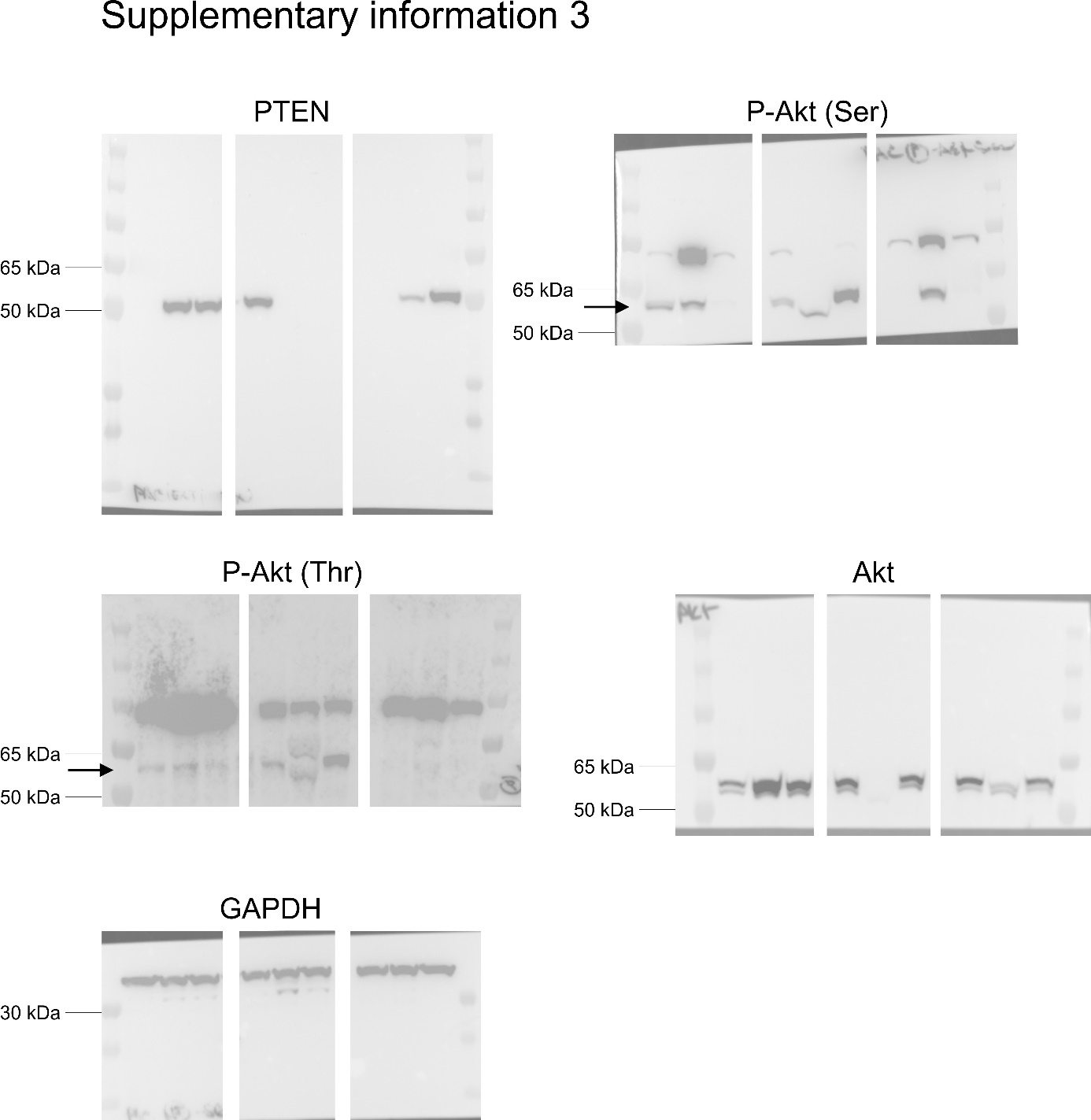


**Supplementary information 3: Full-length gels from Figure 5.**

**
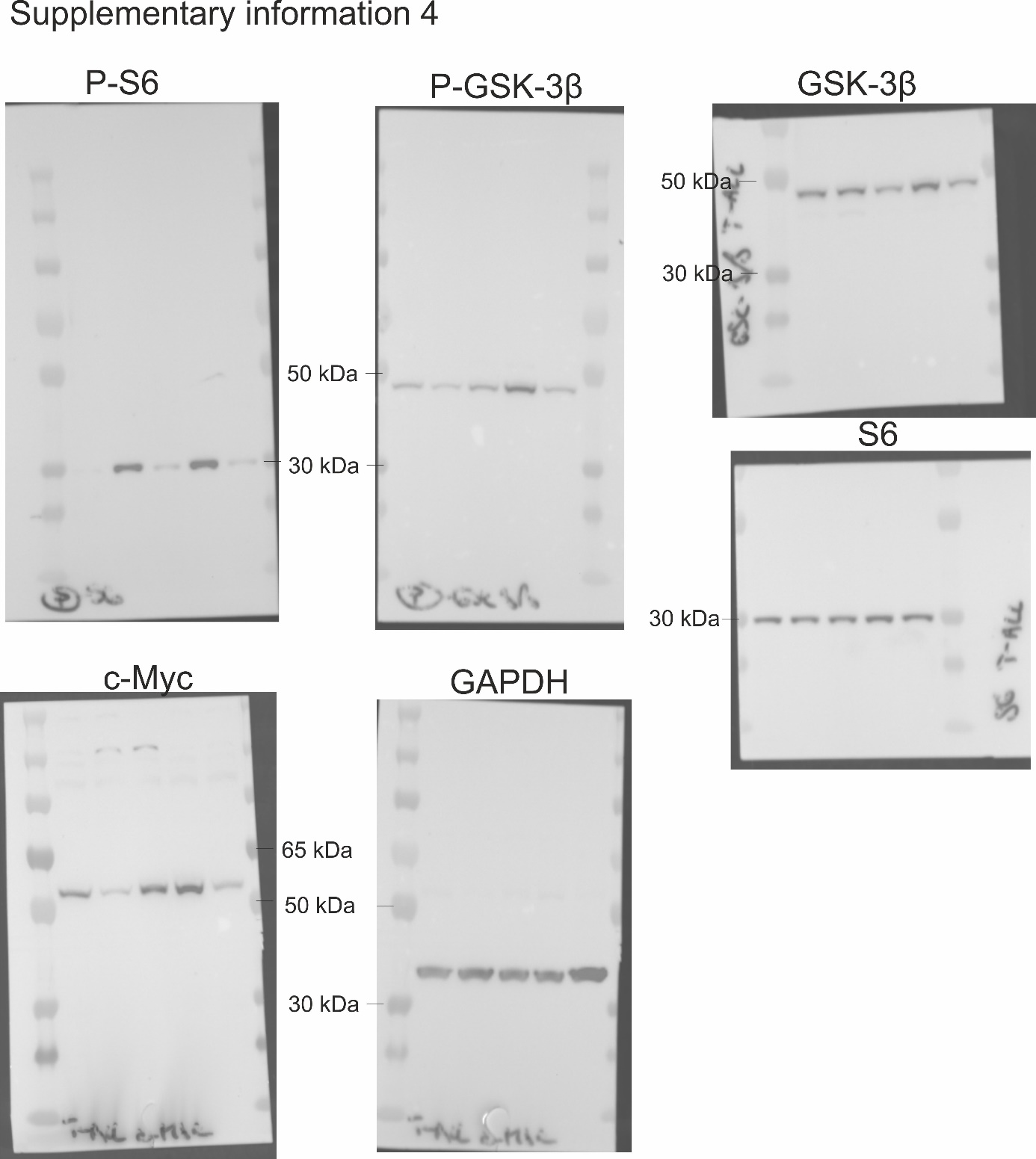
**

**Supplementary information 4: Full-length gels from Supplementary figure 1.**

**
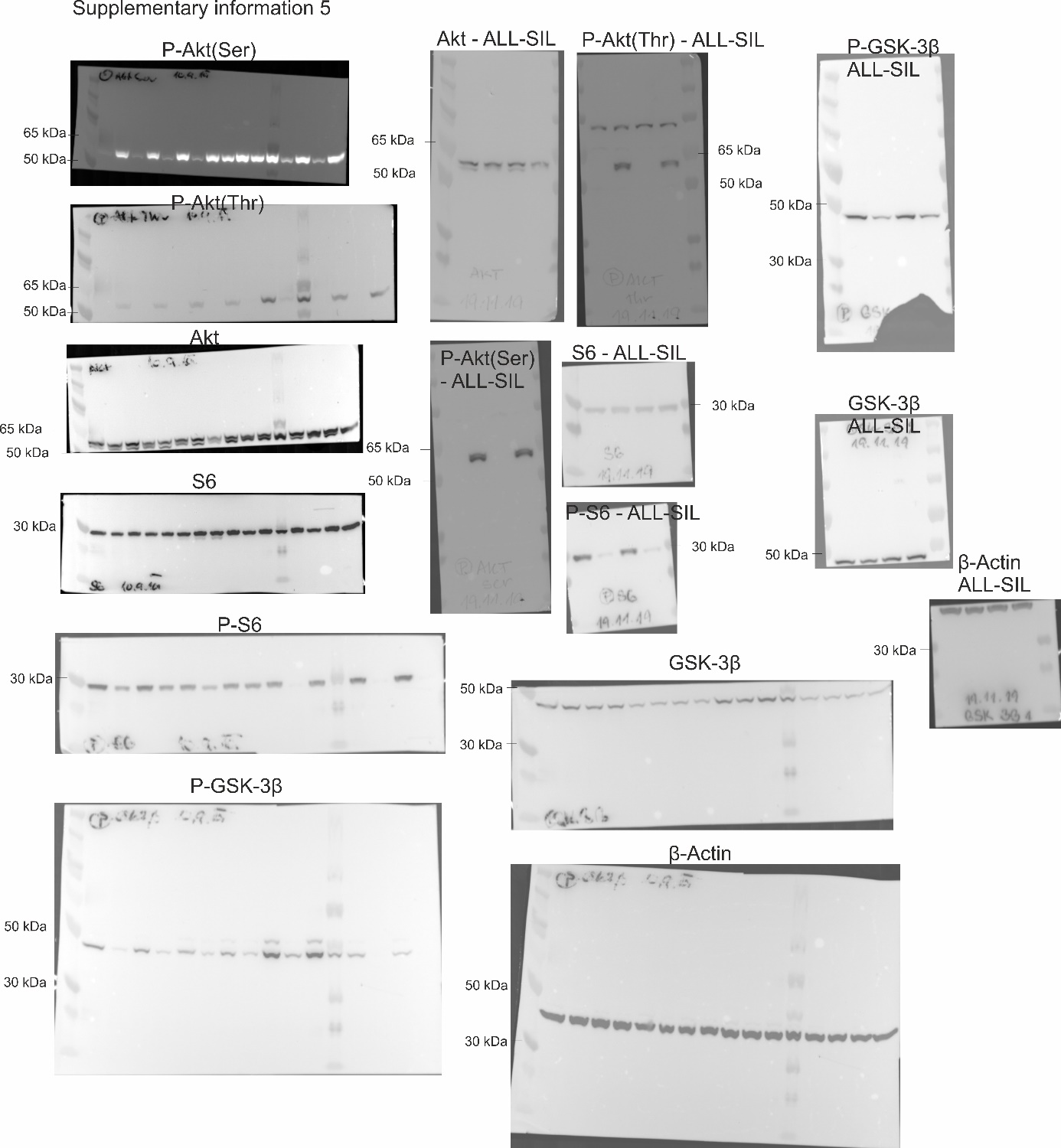
**

**Supplementary information 5: Full-length gels from Supplementary figure 2A.**

**
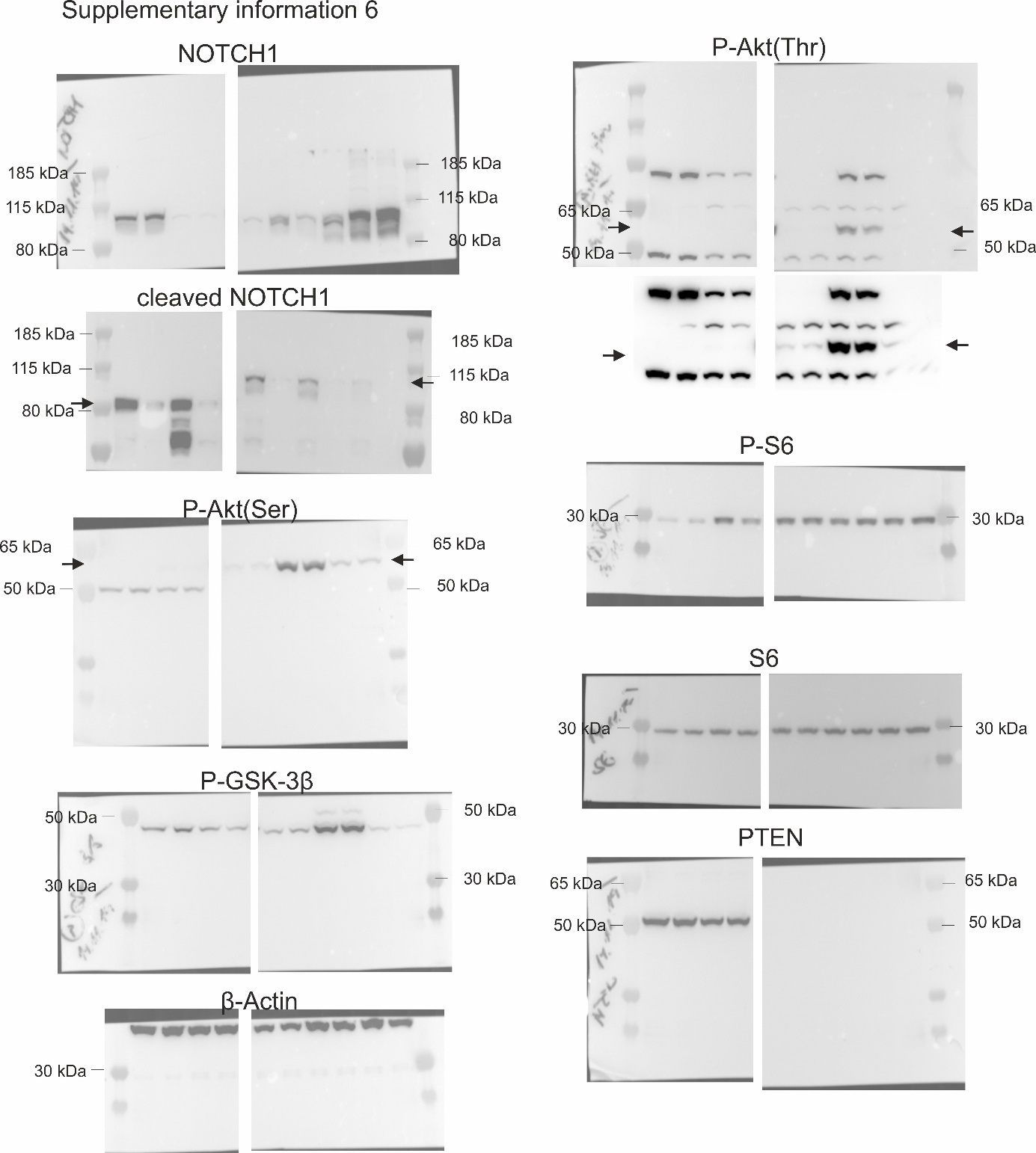
**

**Supplementary information 6: Full-length gels from Supplementary figure 2B.**

**
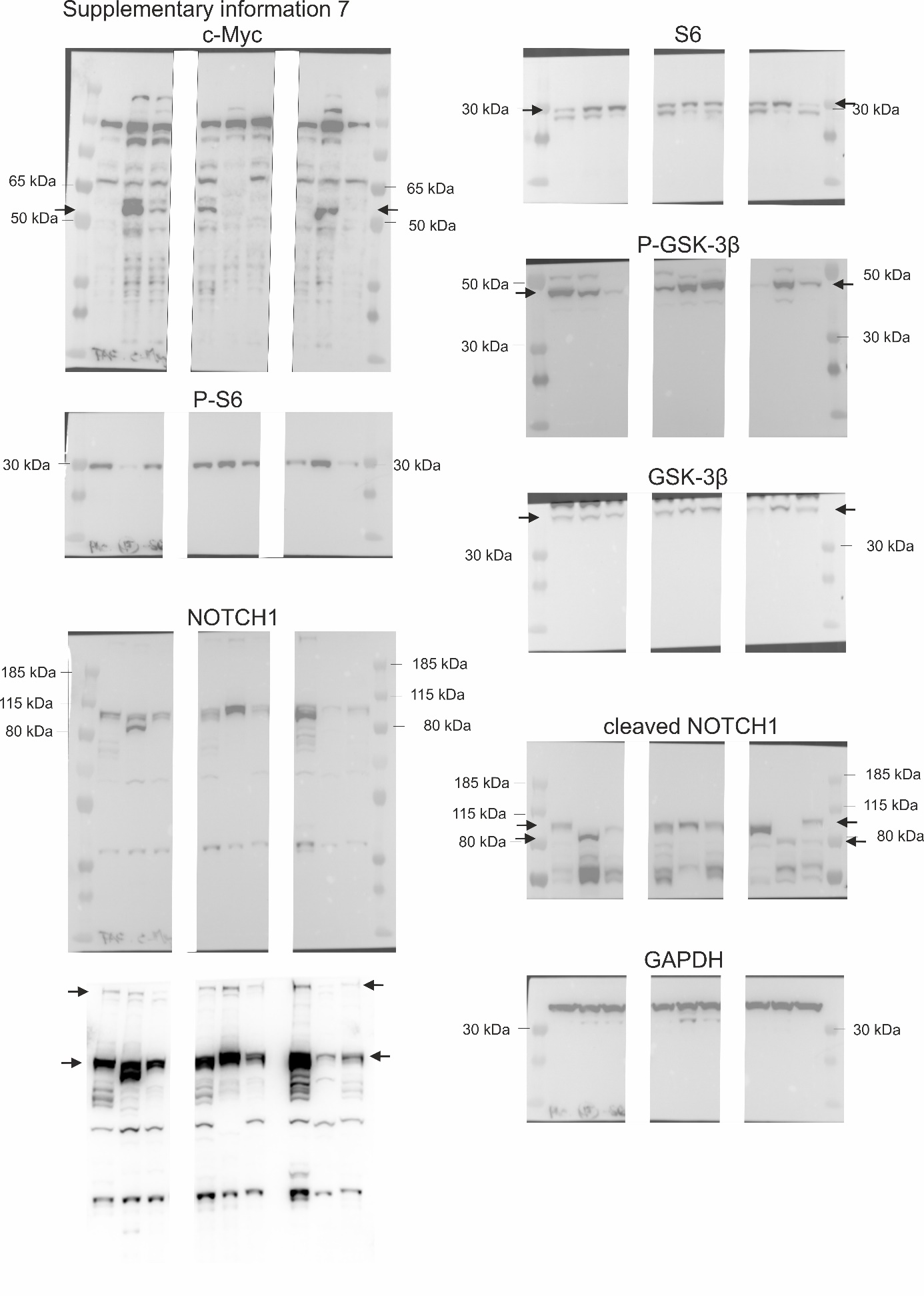
**

**Supplementary information 7: Full-length gels from Supplementary figure 5.**
